# Supplementary material for: Rhinosinusitis Treatment with Cineole: Patient-Reported Quality of Life Improvements from a Non-Interventional, Pharmacy-Based Survey
Source: Medicines (Basel). 2023 Jun 19;10(6):37. doi: 10.3390/medicines10060037 (PMC10301941; doi:10.3390/medicines10060037)
Supplement: Supplementary file 1 [file medicines-10-00037-s001.zip › medicines-2411192-supplementary.pdf]

Supplementary Materials

Table S1. List of decongesting nasal sprays administered during the survey.

Product name	N	%
Aliud Nasenspray AL	3	7.5
Emser Sinusitis Spray	1	2.5
Nasenspray-ratiopharm	9	22.5
nasic Nasenspray	10	25.0
Nasivin Nasenspray	7	17.5
Olynth	1	2.5
Otriven Nasenspray	4	10.0
Rhinospray.Plus	1	2.5
schnupfen endrine	2	5.0
Snup Schnupfenspray	1	2.5
Missing	1	2.5
total	40	100.0

Table S2. Sex distribution of participating subjects administering Cineole capsules or applying a decongesting nasal spray.

Treatment group		N	%
Cineole	female	196	63.2
	male	111	35.8
	intersex	1	0.3
	missing	2	0.6
	total	310	100.0
Nasal spray	female	21	52.5
	male	18	45.0
	intersex	0	0
	missing	1	2.5
	total	40	100.0

Table S3. Age distribution of participating subjects administering Cineole capsules or applying a decongesting nasal spray.

Treatment group	Age range (years)	N	%
Cineole	18-30	71	22.9
	31-45	112	36.1
	46-60	87	28.1
	61-75	29	9.4
	>75	3	1.0
	missing	8	2.6
	total	310	100.0
Nasal spray	18-30	14	35.0
	31-45	4	10.0
	46-60	13	32.5
	61-75	9	22.5
	>75	0	0
	missing	0	0
	total	40	100.0

Table S4. Frequency of rhinosinusitis symptoms prior to and after treatment with Cineole capsules.

frequency	sinus headache / facial pain / facial pressure		blocked or stuffy nose		post-nasal drip		thick nasal discharge		runny nose	
	prior	*after*	*prior*	*after*	*prior*	*after*	*prior*	*after*	*prior*	*after*
N	306	302	308	301	304	301	307	303	305	303
mean	2.22	0.79	2.62	0.95	1.63	0.53	1.67	0.48	2.17	0.95
SD	1.07	0.78	0.90	0.85	1.09	0.73	1.05	0.71	1.07	0.92
median	2	1	3	1	2	0	2	0	2	1
minimum	0	0	0	0	0	0	0	0	0	0
maximum	4	3	4	4	4	3	4	3	4	4

Table S5. Frequency of rhinosinusitis symptoms prior to and after treatment with nasal spray.

frequency	sinus headache/ facial pain/ facial pressure		blocked or stuffy nose		post-nasal drip		thick nasal discharge		runny nose	
	prior	*after*	*prior*	*after*	*prior*	*after*	*prior*	*after*	*prior*	*after*
N	40	40	40	40	40	40	40	40	40	40
mean	1.80	0.83	2.43	1.05	1.40	0.55	1.95	0.63	1.65	1.03
SD	0.94	0.84	0.68	0.68	0.98	0.78	1.06	0.70	0.80	0.83
median	2	1	3	1	1	0	2	0,5	2	1
minimum	0	0	0	0	0	0	0	0	0	0
maximum	4	3	4	3	4	3	4	2	4	3

Table S6. Impact of rhinosinusitis symptoms prior to and after treatment with Cineole capsules.

impact	fatigue		trouble sleeping		concentration problems		performance of normal activities		embarrassment due to nasal symptoms	
	prior	*after*	*prior*	*after*	*prior*	*prior*	*after*	*prior*	*after*	*prior*
N	306	302	306	301	307	303	302	302	303	300
mean	2.19	1.15	2.08	1.06	1.93	0.92	1.65	0.69	1.17	0.50
SD	0.94	0.89	1.07	0.93	0.99	0.84	1.01	0.89	1.07	0.79
median	2	1	2	1	2	1.00	2	0	1	0
minimum	0	0	0	0	0	0	0	0	0	0
maximum	4	4	4	4	4	4	4	4	4	3

impact	being frustrated		irritability		sadness		thoughts on nasal symptoms	
	prior	*after*	*prior*	*after*	*prior*	*prior*	*after*	*prior*
N	303	299	308	302	305	300	307	303
mean	1.19	0.52	1.36	0.60	0.78	0.34	1.18	0.44
SD	1.07	0.79	1.07	0.8	0.935	0.63	1.13	0.72
median	1	0	1	0	1	0	1	0
minimum	0	0	0	0	0	0	0	0
maximum	4	3	4	3	4	3	4	4
